# A Workflow to Produce a Low-Cost In Vitro Platform for the Electric-Field Pacing of Cellularised 3D Porous Scaffolds

**DOI:** 10.1021/acsbiomaterials.3c00756

**Published:** 2023-08-02

**Authors:** Matteo Solazzo, Michael G. Monaghan

**Affiliations:** † Department of Mechanical, Manufacturing and Biomedical Engineering, Trinity College Dublin, 152−160 Pearse Street, Dublin 2, Ireland; ‡ Advanced Materials and BioEngineering Research (AMBER) Centre at Trinity College Dublin and the Royal College of Surgeons in Ireland, Dublin 2, Ireland; § CÚRAM, Centre for Research in Medical Devices, National University of Ireland, Galway, Newcastle Road, Galway H91 W2TY, Ireland; ■ Trinity Centre for Biomedical Engineering, 152-160 Pearse Street, Dublin 2, Ireland

**Keywords:** biomaterials, electroconductive biomaterials, electrostimulation, polymer chemistry, organoid generation

## Abstract

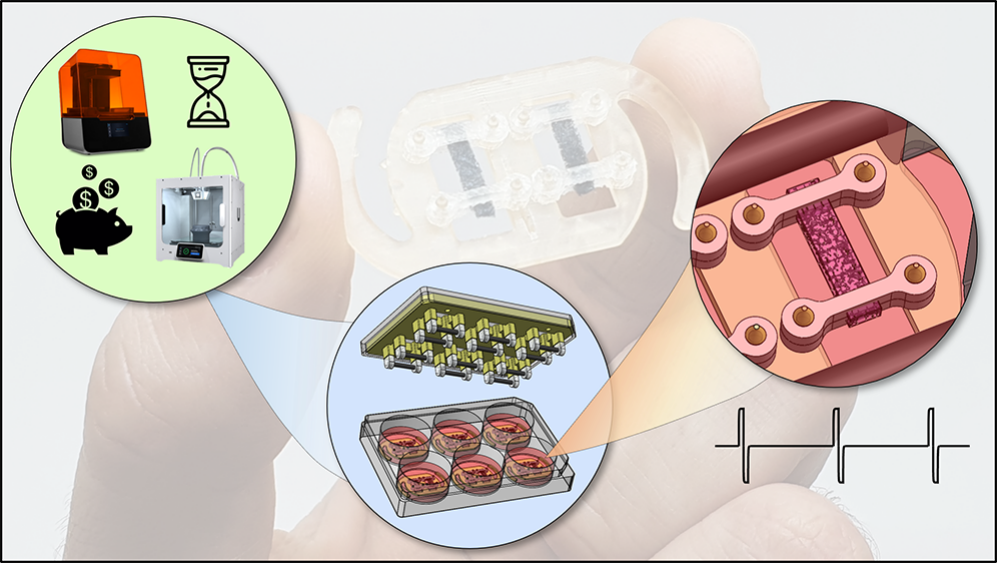

Endogenous electrically mediated signaling is a key feature of most native tissues, the most notable examples being the nervous and the cardiac systems. Biomedical engineering often aims to harness and drive such activity in vitro, in bioreactors to study cell disease and differentiation, and often in three-dimensional (3D) formats with the help of biomaterials, with most of these approaches adopting scaffold-free self-assembling strategies to create 3D tissues. In essence, this is the casting of gels which self-assemble in response to factors such as temperature or pH and have capacity to harbor cells during this process without imparting toxicity. However, the use of materials that do not self-assemble but can support 3D encapsulation of cells (such as porous scaffolds) warrants consideration given the larger repertoire this would provide in terms of material physicochemical properties and microstructure. In this method and protocol paper, we detail and provide design codes and assembly instructions to cheaply create an electrical pacing bioreactor and a Rig for Stimulation of Sponge-like Scaffolds (R3S). This setup has also been engineered to simultaneously perform live optical imaging of the in vitro models. To showcase a pilot exploration of material physiochemistry (in this aspect material conductivity) and microstructure (isotropy versus anisotropy), we adopt isotropic and anisotropic porous scaffolds composed of collagen or poly(3,4-ethylene dioxythiophene):polystyrenesulfonate (PEDOT:PSS) for their contrasting conductivity properties yet similar in porosity and mechanical integrity. Electric field pacing of mouse C3H10 cells on anisotropic porous scaffolds placed in R3S led to increased metabolic activity and enhanced cell alignment. Furthermore, after 7 days electrical pacing drove C3H10 alignment regardless of material conductivity or anisotropy. This platform and its design, which we have shared, have wide suitability for the study of electrical pacing of cellularized scaffolds in 3D in vitro cultures.

## 1 Introduction

Bioreactors can recapitulate biophysical stimuli, scale up growing cultures to generate sufficient numbers of therapeutic cells, growth factors, proteins, and hormones and study physiological responses ex vivo.^1^ Such stimuli include strain and stress, pressure, flow, and the subject of this paper: electrical pacing. Pacing is commonly used with engineered heart tissues derived from primary cardiomyocytes and cardiomyocytes derived from pluripotent stem cell sources,^2^ neural applications in the case of neuronal cultures,^3^ and skeletal muscle cultures.^4^ Aside from these “traditional” tissues, endogenous electrically mediated signaling pervades and is present in all aspects of cellular biology with electrical pacing being explored in nontraditional tissues such as skin,^5^ bone,^6^ and immune cells^7^ more recently.

Often, electric field-based systems stimulate cells embedded self-assembling in fibrin^8^ or collagen^2^ gels, in the form of tubules,^8^ rings,^9^ or patches.^10,11^ For example, engineered heart tissues are often incorporated into bioreactors capable of delivering diverse cues (e.g., shear rate, mechanical constrain, electrical stimulation), and these stimuli have been shown to support the maturation of induced pluripotent derived cardiomyocyte (iPSC-CMs) cells toward more adult-like phenotypes with functionality.^12^ Electrical pacing can also synchronize cell beating with an effect similar to the action potential generated in the sinoatrial node, and it can also be used to tune the beating frequency of the engineered heart tissues itself,^13,14^ study inherited genetic defects in engineered heart tissues,^15^ and act as a promising therapy to reverse symptoms of acquired arrhythmia.^16^

An underexplored approach in engineering tissues which are then subjected to electric field stimulation is seeding cells onto a pre-existing, preformed scaffold, possessing predefined geometry and stiffness.^17^ Due to simplicity of fabrication and the option to embed and mix cells prior to gelation, many researchers have focused on the scaffold-free self-assembling method.^10^ However, the self-assembling method is limited in the suitable materials and poses limitations in the repertoire of material properties (stiffness, chemistry, microarchitecture, and conductivity).

The burgeoning progress of electroconductive biomaterials, especially conjugated polymers (CPs), has demonstrated potential to assist the regeneration of the heart wall after myocardial infarction (MI),^18−20^ and it is hypothesized that such electroconductive platforms could provide enhanced electrophysiological support when compared to biologically derived materials.^21^ While their use is gathering momentum,^22^ the inclusion of electroconductive materials into engineered tissues is yet to be fully exploited and warrants inclusion in electric field stimulation setups.

The use of porous scaffolds with predefined microarchitecture could directly influence cell response, in particular, their spatial orientation. Anisotropic tissue microarchitecture can guide the alignment of cells along the direction of contraction and their fusion into structures.^23,24^ In addition to anisotropic ordering of materials, stiffness is another physical property known to play a significant role in promoting the differentiation of progenitor cells toward a desired progeny.^25^

In this work, we describe the development of a custom electric-field bioreactor system for use with prefabricated porous scaffolds for three-dimensional (3D) electric-field stimulation. As a demonstrative example, we apply electroconductive scaffolds based on crystallized poly(3,4-ethylene dioxythiophene):polystyrenesulfonate cross-linked with (3-glycidyloxypropyl) trimethoxysilane (PEDOT:PSS-GOPS) as 3D substrates for in vitro models based on our extensive experience with this material. Scaffolds manufactured with this material have native tissue-like electrical conductivity, which is expected to enhance the cellular response to electric pacing.^26^ This in-house bioreactor is capable of applying electrical pacing to monolayer cell cultures as well as to 3D constructs, and its construction and design is documented in detail in this methods paper. Furthermore, we engineered and manufactured a Rig for the Stimulation of Sponge-like Scaffolds (R3S); an in vitro platform designed to maintain scaffolds suspended reliably in culture media while enabling free uniaxial contraction of the cell laden constructs and simultaneously providing optical accessibility for both standard and inverted microscopy. This is achieved using custom-made grips incorporated into this platform, the stiffness of which can be tuned toward the application being studied. We report validation of this system using a C3H10 cell line and demonstrate that scaffold morphology and electrical pacing play a synergistic role in the proliferation and metabolism of cells. Moreover, C3H10 cells preferentially aligned along the direction of the electric field, even in the absence of an anisotropic substrate.

Detailed methods on the fabrication and assembly of this bioreactor system, including printing codes where applicable, are shared. The information disseminated in this paper will enable researchers to develop their own electric-field bioreactor assembly system to suit a diverse number of scaffold-based cultures and various other potential applications.

## 2 Experimental Section

### 2.1 Modeling and 3D Printing

Throughout the project, 3D models were designed and then fabricated via rapid prototyping (Table 1). All software design was performed with Solidworks while 3D printing was achieved with either Original Prusa i3MK3S (Prusa Research), Ultimaker 3 (Ultimaker BV), or stereolithography (SLA) printer Form 3 (Formlabs). These files are available in the Supporting Information.

**Table 1:** Specifications of 3D Printed Components

Part name	Printer	Material^a^	File reference
Mold for elastomeric bars	Original Prusa i3MK3S/Ultimaker 3	PLA	01_ElastomericBars_Mold_BOTTOM.STEP 02_ElastomericBars_Mold_MIDDLE.STEP 03_ElastomericBars_Mold_TOP.STEP
Mold for carbon electrodes	Original Prusa i3MK3S/Ultimaker 3	PLA	04_ElectrodesEmbedding_Mold_BOTTOM.STEP 05_ElectrodesEmbedding_Mold_TOP.STEP
Lid Adapter	Original Prusa i3MK3S/Ultimaker 3	PLA	06_LidAdapter.STEP
R3S	Form 3	Dental LT Clear Resin	07_R3S.STEP
Mold for Seeding chambers	Original Prusa i3MK3S/Ultimaker 3	PLA	08_SeedingChambers_Mold.STEP

a PLA = polylactic acid.

### 2.2 Processing of 3D Sponge-Like Scaffolds

One can use their own scaffolds for incorporation into the R3S, but we describe the fabrication of the scaffolds used in this study briefly. We recommend users fashion their final construct into the dimensions described at the end of this section.

In this study porous sponge-like scaffolds were generated using either collagen type I slurry or PEDOT:PSS-based dispersion, with collagen type I as the natural extracellular matrix-based sponge material, resulting in an electrically insulant material, and PEDOT:PSS scaffolds as the electroconductive control which we have worked with extensively in another study.^27^

Collagen type I was isolated from porcine tendons which are extensively described.^27^ The isolated collagen type I was then solubilized in 0.1 M acetic acid to a final concentration of 15 mg/mL. PEDOT:PSS 1.3 wt.% dispersion in water (Sigma-Aldrich, Ireland) was mixed with 3 v/v% GOPS (average *M*_n_ 236.34, Sigma-Aldrich, Ireland). PEDOT:PSS blends were vortexed for 30 s, sonicated for 30 min, and filtered with a 0.45 μm polyvinylidene diflouride (PVDF) syringe filter to remove aggregates.

Porous 3D sponge-like scaffolds were created through lyophilization using a freeze-dryer (FreeZone, Labconco Corporation, USA). Isotropic scaffolds were produced using standard cell culture multiwell plates, while a custom-made mold containing a bottom stainless-steel layer and a top polydimethylsiloxane (PDMS) (SYLGARD 184, Farnell, Ireland) was fabricated to induce ice crystal alignment by virtue of the different thermal conductivities of the two materials, ultimately resulting in anisotropic scaffolds.^28^ Freezing was performed at −40 °C for 1 h, after which the temperature was raised to −10 °C at a rate of 1 °C per minute, held for 18 h at a vacuumed environment of 0.2 mbar, ramped to 20 °C at a rate of 1 °C per minute, and finally held for 2 h.

PEDOT:PSS-GOPS scaffolds underwent annealing treatment in a vacuum oven at 140 °C for 1 h and were crystallized as previously reported.^27^ Briefly, PEDOT:PSS-GOPS samples, with both isotropic and anisotropic architecture, were submerged into a bath of 100% sulfuric acid for 15 min, then washed multiple times using deionized water.

After freeze-drying, collagen scaffolds were cross-linked with *N*-(3-dimethylaminoproypl)-*N*-ethylcarbodiimide hydrochloride (EDC) (E1769, Sigma-Aldrich) and *N*-hydroxysuccinimide (NHS) (130672, Sigma-Aldrich). A 5:5:1 molar ratio of EDC/NHS/carboxyl groups within the collagen scaffolds was added to pure ethanol, and the pH was adjusted to between 5.3 and 5.5.^29^ Cross-linking took place under rocking for 18 h; samples were then rehydrated and lyophilized one more time.

Scaffolds were finally sectioned at 1 mm thickness with a vibratome (VT 1200S, Leica. Germany). Regular rectangular strips 9 mm long and 2 mm wide were manually cut using a razor blade. Scaffolds were sterilized with multiple washings in 70% ethanol and exposure to UV light followed by preconditioning in standard growth media.

### 2.3 Fabrication of Pacing Bioreactor

**Figure 1:**
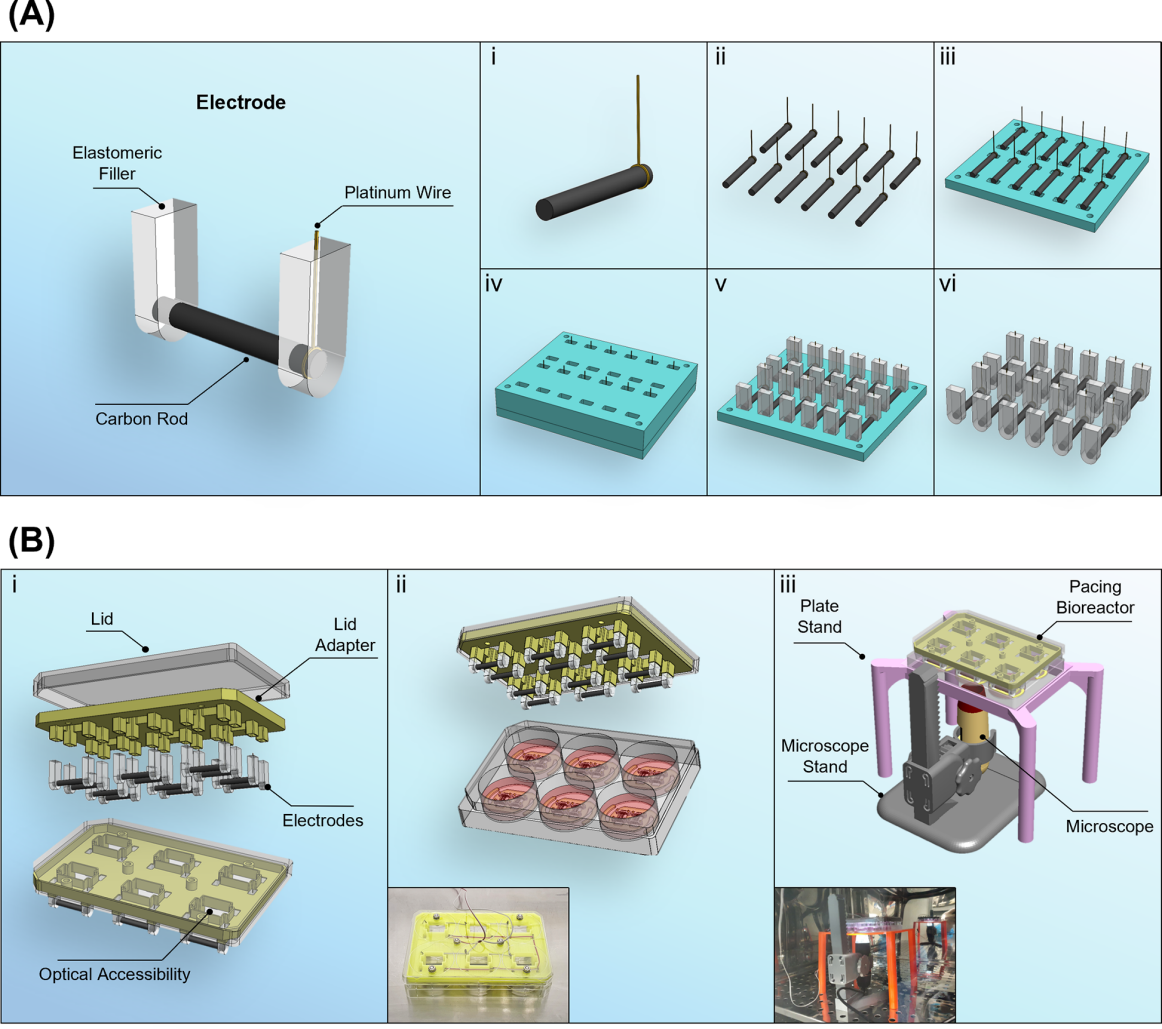
Design and manufacturing of the electrical pacing bioreactor. (A) Schematic representation of a carbon electrode. (i–vi) Fabrication sequence of the electrodes. (i) A platinum–iridium wire is tightened to one extremity of a carbon bar. (ii) Step i is repeated until achievement of 12 elements. (iii) Bars are inserted into the bottom component of the mold. (iv) The top component of the mold is secured, and pristine PDMS is poured into each hole. (v) Polymerization is completed, and the top component of the mold can be removed. (vi) Once the bottom component is removed, 12 carbon electrodes are achieved. (B) Overview of the electrical pacing bioreactor. (i) Exploded and collapsed views of the individual components of the electrical pacing bioreactor. (ii) Schematic and picture of the custom-made cell culture pacing setup. (iii) Schematic and picture of the custom-made setup for the observation and monitoring of scaffolds. Scale bar: A = 1 cm.

To electrically pace cells upon scaffolds, we can deliver electrical stimulation via the custom-made bioreactor described. We conceptualised and manufactured the system with the use of inexpensive rapid prototyping and standard poly(lactic acid) (PLA), gathering inspiration from existing models.^12,30^ A schematic representation of the setup is shown in Figure 1. A set of 12 22 mm long cylindrical carbon electrodes are cut from a 3 mm large carbon rod (Goodfellow Cambridge Ltd., UK) and a 1 mm large hole drilled at one end only through the center point of the cylinder radius, through which a platinum–iridium wire (Advent Research Materials Ltd., UK) is tied. A mold, consisting of a top and a bottom component (Figure S1), is 3D printed to allocate 12 electrodes, and, once the two components are sealed together, PDMS Sylgard 184 is introduced in the mold. After polymerization as per the instructions for Sylgard, the mold is opened, and the electrodes can be extracted (Figure 1.A and Video 1). The silicone closures at both extremities of each carbon bar allow permanent securing of the platinum–iridium wire and avoidance of any liquid infiltration, while also providing an anchorage system to the *lid adapter.* This 3D printed component interfaces between the electrodes and the plate lid (Figures 1.Bi and S2 and Video 2); it holds 6 pairs of carbon rod electrodes kept at 15 mm distance between each other and suitable to pace a 6-well plate. This component is designed to have slots for six screws to secure it to a 6-well plate lid, with openings in the area in between the electrode pairs for optical accessibility using both upright and inverted microscopes. A 5 mm depth space between this interface and the lid accounted for the wiring. Electrodes are connected with copper wire to have a configuration in parallel as represented in Figure S3. The use of a parallel configuration and the fabrication of the electrodes via mold-injection are designed to provide an equal electrical response for all six pairs of electrodes. The source ground and positive wires connect externally to a breadboard, where a waveform generator (RSDG2000X, Radionics) and an oscilloscope (RSDS 1052 DL+, Radionics) are also connected. The oscilloscope serves to validate the signal being generated by the waveform generator. Before and after every experiment, the bioreactor should be sterilized with multiple cycles of incubation in 70% (v/v) ethanol followed by sterile deionized water, and last being exposed both internally and externally to UV light. Electrodes should be inspected and tested before each stimulation regime for corrosion or damage and validated using an oscilloscope. While this study applied short daily regimes of one hour of electrical stimulation, longer-term stimulation regimes should be monitored closely. The reader is recommended to refer to the work of Tandon et al. in such cases.^31^

### 2.4 Fabrication and Tensile Testing of Elastomeric Bars

**Figure 2:**
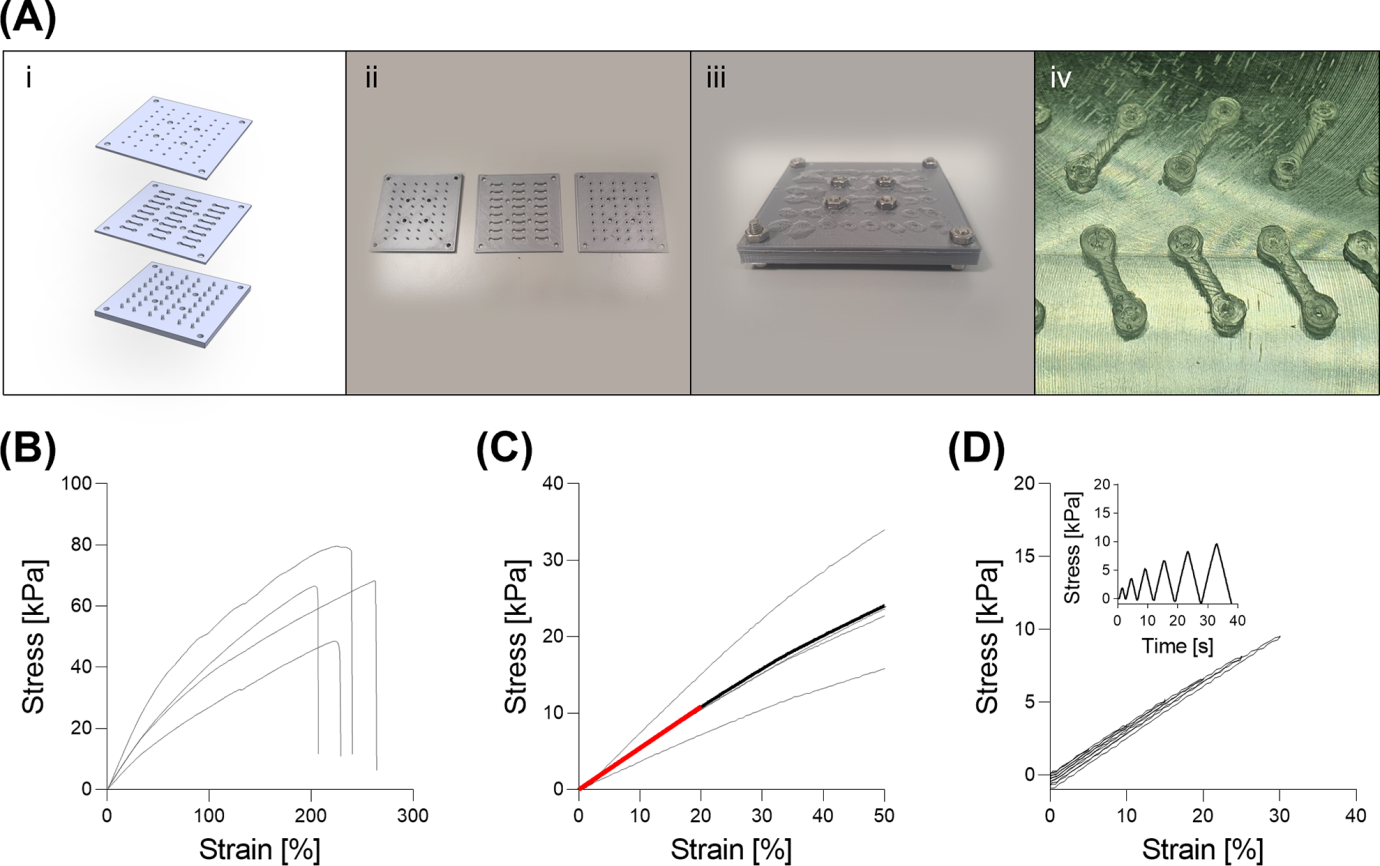
(A) Manufacturing of PDMS elastomeric bars. (i) Illustrations of bottom, middle, and top components of the mold. (ii) 3D printed components of the mold. (iii) Assembled mold that has been filled with pristine PDMS. (iv) A set of manufactured PDMS elastomeric bars. (B–D) Mechanical characterization of PDMS bars via uniaxial tensile test. (B) Tension was maintained until rupture. (C) Tension in the range 0–50% strain with mean linear interpolation in red. (D) Stress–strain and stress-time curves represent a standard response within cycles from 5% to 30% strain. Scale bar: A.iv = 1 cm.

Elastomeric bars are designed in dumbbell-like shapes and are placed in pairs at each extremity of the porous scaffolds in a sandwich-like fashion. These bars are casted within a multicomponent custom-made mold (Figure 2.Ai-iii) with square cross section with 1 mm long sides (Figure 2.Aiv). PDMS blended as a 1:5 ratio of Sylgard 184 and Sylgard 527 is recommended, to generate a material strong enough to keep the scaffolds in place, but with appropriate flexibility to be deflected by contracting scaffolds. The blending of Sylgard can be optimized according to the user’s application by varying of ratios.

It is recommended that uniaxial tensile tests are performed on these elastomeric bars to characterize their stiffness. For the purpose of this study and demonstration, a cyclic sequence with increasing strain from 5% to 30% by 5% increments was performed to assess the absence of hysteresis under dynamic conditions. Then, a ramp until failure followed to verify the rupture limit (Figure 2). Bars had a mean elastic modulus of 54.3 ± 15.7 kPa, and no evidence of hysteresis was observed in the tested range.

### 2.5 R3S: Rig for the Stimulation of Sponge-Like Scaffolds

For the stimulation and optical monitoring of 3D sponge-like scaffolds, a specific rig that could maintain scaffolds in a stable position is provided. In addition, for the application of such a system to tissue engineering, this rig would not impede contraction of scaffolds generated by beating skeletal muscle myocytes or cardiomyocytes and/or provide the necessary strain boundary conditions warranted in other applications.

**Figure 3:**
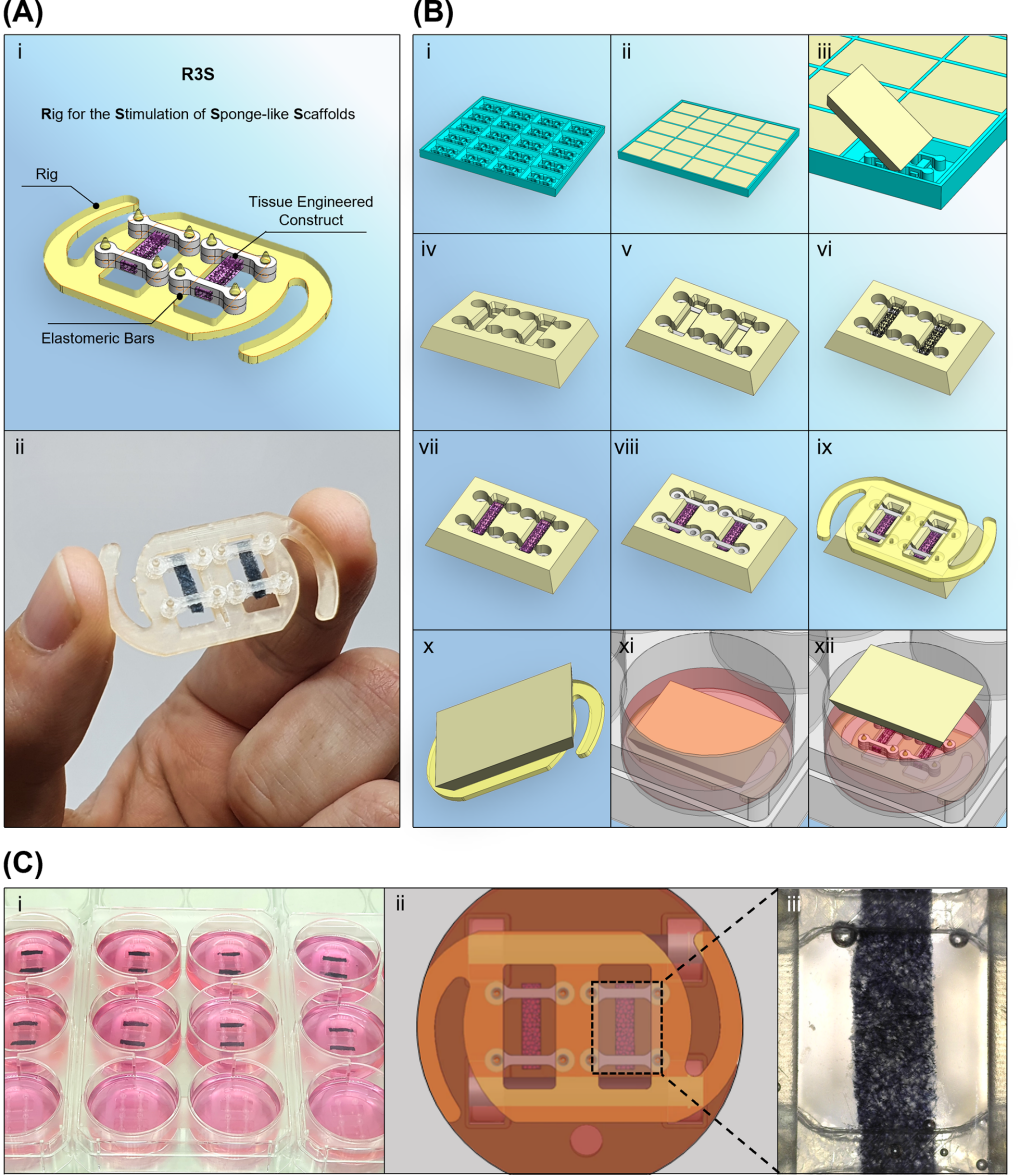
(A) (i) Schematic and (ii) picture of R3S (Rig for the Stimulation of Sponge-like Scaffolds). (B) (i-iv) Sequence of preparation of seeding chambers. (v-xii) Sequence of assembly of R3S. (C) Assembled cell culture setup, showing (i) a picture of a set of plates with R3S, (ii) a schematic of the view from the bottom of the plate with focus on a single well, and finally (iii) a picture obtained with the microscope camera focusing on a single scaffold.

The system presented in Figure 3.A is composed of a main body and four elastomeric bars. The main body (Figure S4 and Video 3) was rapid-prototyped via SLA and designed to accommodate two 3D scaffolds to be kept parallel with the electric field generated by the bioreactor that can be observed from optically accessible chambers placed underneath. On the side and at the extremities of each of these chambers, a pillar with a sharp tip anchors the elastomeric bars.

### 2.6 Cell Culture and Evaluation of Biocompatibility

After fabrication, optimization, and assembly of the bioreactor and R3S platform described and shared in this manuscript, we next moved to trial its operation in a proof-of-concept study. C3H10 mouse embryonic fibroblasts (ATCC CCL-226TM) were cultured in growth media prepared with Dulbecco’s Modified Eagle’s Medium (DMEM) low glucose (Sigma-Aldrich) containing 10 v/v% fetal bovine serum (FBS) (Gibco by Life Technologies) and 2 v/v% Penicillin Streptomycin (Pen-Strep) (Sigma-Aldrich) at 37 °C with 5 v/v% CO_2_. Cells between passages 12 and 15 were used. 3D scaffolds were sterilized immediately prior to use undergoing multiple cycles of incubation in 70 v/v% ethanol under UV light, followed by sterile deionized water, and finally incubated overnight in growth media for 24 h.

### 2.7 Seeding Protocol and Validation of Pacing Bioreactor with C3H10

To facilitate accurate assembly of the rig and for consistency between replicates, while also confining the cell seeding volume, we designed and recommended cell seeding chambers for use with this bioreactor system. These are achieved by the addition of sterile 2 w/v% agarose solution into a 3D printed mold specifically designed to fit rectangular sponge-like scaffolds with dimensions of 2 × 1 × 9 mm and four elastomeric bars per scaffold (Figures 3.A and S5 and Video 4). The agarose solution solidifies within minutes, and such an approach is adopted in a number of studies in the tissue engineering context, to gain a high cell seeding yield.^32,33^

For validation of the pacing bioreactor and of R3S, C3H10 were seeded as illustrated in Figure 3.B and Video 5. Specifically, two elastomeric bars are set in each compartment of the seeding chamber. Scaffolds are placed, and any excess liquid is removed. In this study, the cell suspension is mixed in equal amount with Matrigel to reach a final seeding volume of 20 μL and 250 000 cells and pipetted onto the dry scaffold. Matrigel allows faster incorporation of cells with the scaffold, although users may adopt and optimize other matrix components to achieve this. After an incubation of 30 min at 37 °C, to allow the transition from liquid to gel of the media/Matrigel, two more elastomeric bars should be placed on top of the scaffold in a sandwich-like fashion. At this stage, the main body of R3S is pressed into the seeding chamber, piercing the agarose and locking the elastomeric bars and scaffolds in position. The setup is then flipped and transferred to a 6-well plate prefilled with culture media. After one more hour of incubation, the agarose seeding chamber can be removed (Figure 3.C and Video 6). Here, beginning from day 3, C3H10 cells were paced with a biphasic pulsatile regime on the order of ±2.5 V, 2 ms pulse, 2 Hz. The stimulation regime and duration can be adopted according to the user needs. Here, a daily 1 h long conditioning session was carried out until day 7 (Video 7).

### 2.8 Live/Dead Assay

Cell viability was assessed using a live/dead assay implementing a solution of 2 μL/ml of Ethidium Homodimer and 0.5 μL/ml of Calcein (Cambridge Bioscience) in PBS. This solution was incubated at 37 °C for 1 h and afterward washed three times with PBS. Samples were kept at 37 °C until imaged. Imaging was performed with a Leica SP8 scanning confocal microscope (Leica Microsystems, Germany). For quantification, a minimum of 3 pictures per experimental replicate were taken and subsequently analyzed with ImageJ. Pictures were obtained as resultant z-projection of z-stack with different z-step and depth of penetration from the surface. Specifically, a z-step size of 25 μm and depth of 125 μm were used. Cell viability was defined as the ratio of live cells over the total cell number (%), while the live cell density was obtained as ratio between live and total cells normalized by the region of interest (ROI) area (cells/mm^2^).

### 2.9 DAPI/Phalloidin Fluorescent Staining

Cell spreading was evaluated by using cytofluorescent staining. After three washings in PBS, samples were fixed in 4% (w/v) paraformaldehyde for 60 min at room temperature. Following three more washings in PBS, samples were incubated within a working dye solution prepared with 1 μL/ml phalloidin (Santa Cruz Technology, USA) and 4′,6-diamidine-2′-phenylindoledihydrochloride (DAPI, 1 mg/mL, Sigma-Aldrich, Ireland), to highlight filamentous actin (f-actin) of the cell cytoskeleton and cell nuclei, respectively. Micrographs were obtained using a Leica SP8 scanning confocal microscope (Leica Microsystems, Germany), and image processing for nuclei orientation was performed with custom-made scripts in Matlab.

### 2.10 AlamarBlue Metabolic Assay

To evaluate the cell condition, a standard AlamarBlue metabolic assay was performed. After removal of cell culture media and one washing with PBS, an AlamarBlue working solution containing AlamarBlue reagent was added to the culture and incubated for 1 h. Media were gently mixed with pipet and incubated for 1 h; afterward, the media were mixed again and moved to a 96-well plate for analysis. Results have been quantified in terms of “Reduced%” of resazurin into resorufin via cells aerobic respiration.^34^

### 2.11 Picogreen Assay

A biochemical assay was used to identify DNA content using a Picogreen assay. At specific time points, samples were washed in PBS and frozen at −80 °C until the moment of the assay. Samples were digested in a papain-based enzymatic solution in dynamic conditions for 18 h, and analysis was then performed according to the provided standard protocol.

### 2.12 Statistical Analysis

Statistical analysis was performed using GraphPad Prism 9 (GraphPad Software, USA). Where appropriate a one-way or two-way analysis of variance (ANOVA) followed by Tukey’s multiple comparison was utilized. If not otherwise specified, results are presented as mean ± standard deviation, and differences are considered as statistically significant for *p* < 0.05.

## 3 Results and Discussion

**Figure 4:**
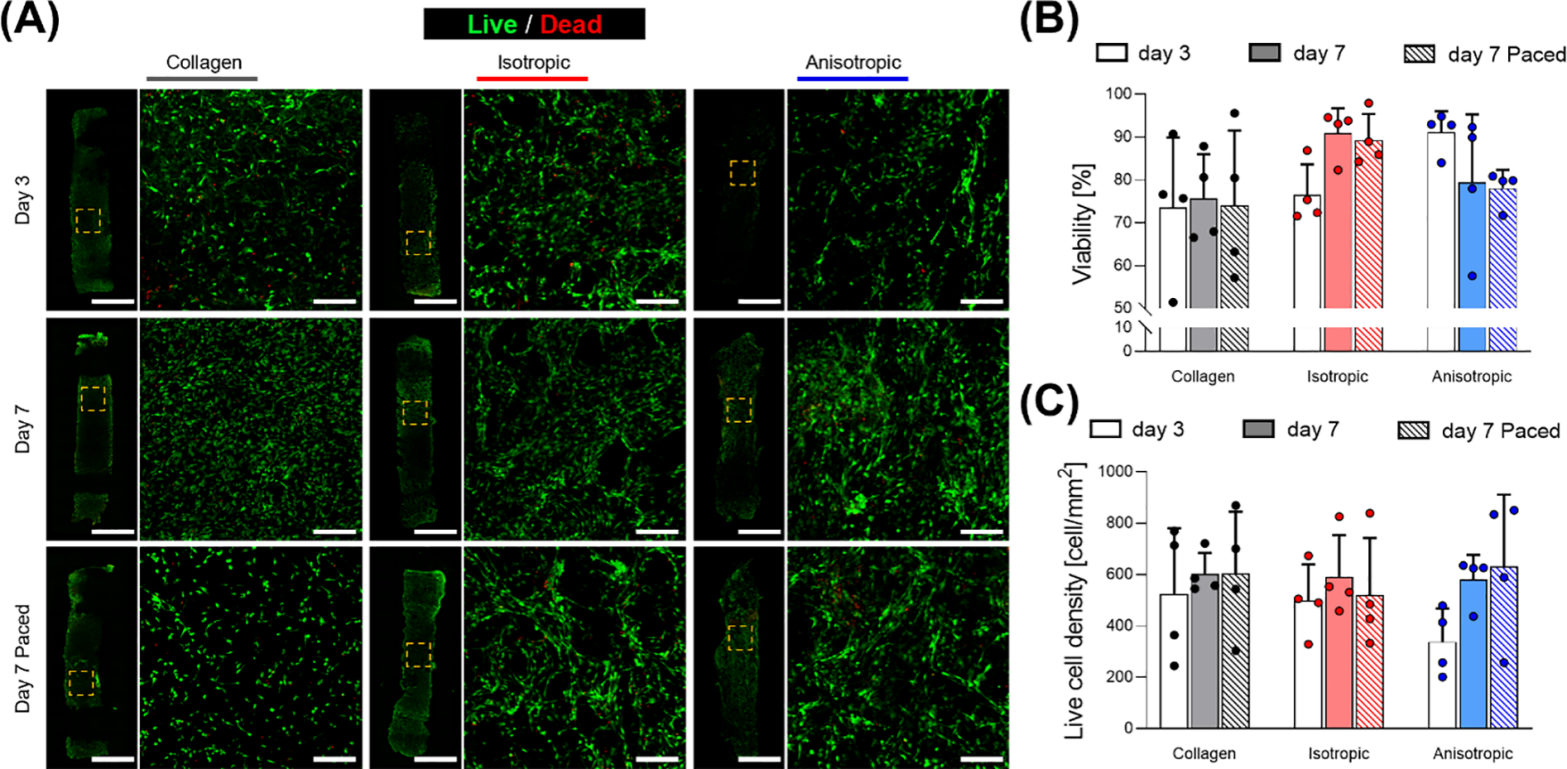
Effects of pacing C3H10 cells in scaffolds on viability. (A) Micrographs from confocal microscope fluorescent staining for live/dead of CH310 cells at days 3, 7 with and without pacing. (B, C) Quantification of viability (extracted from live/dead staining) at days 3 and 7 with and without pacing quantified as viability and alive cell density (*n* = 4). Scale bars: A = 2 mm; A insets = 200 μm. Bar graphs demonstrate the mean with error bars representing standard deviation. Data values are presented as associated points. Statistical significance was performed using two-way ANOVA with Tukey’s posthoc test.

**Figure 5:**
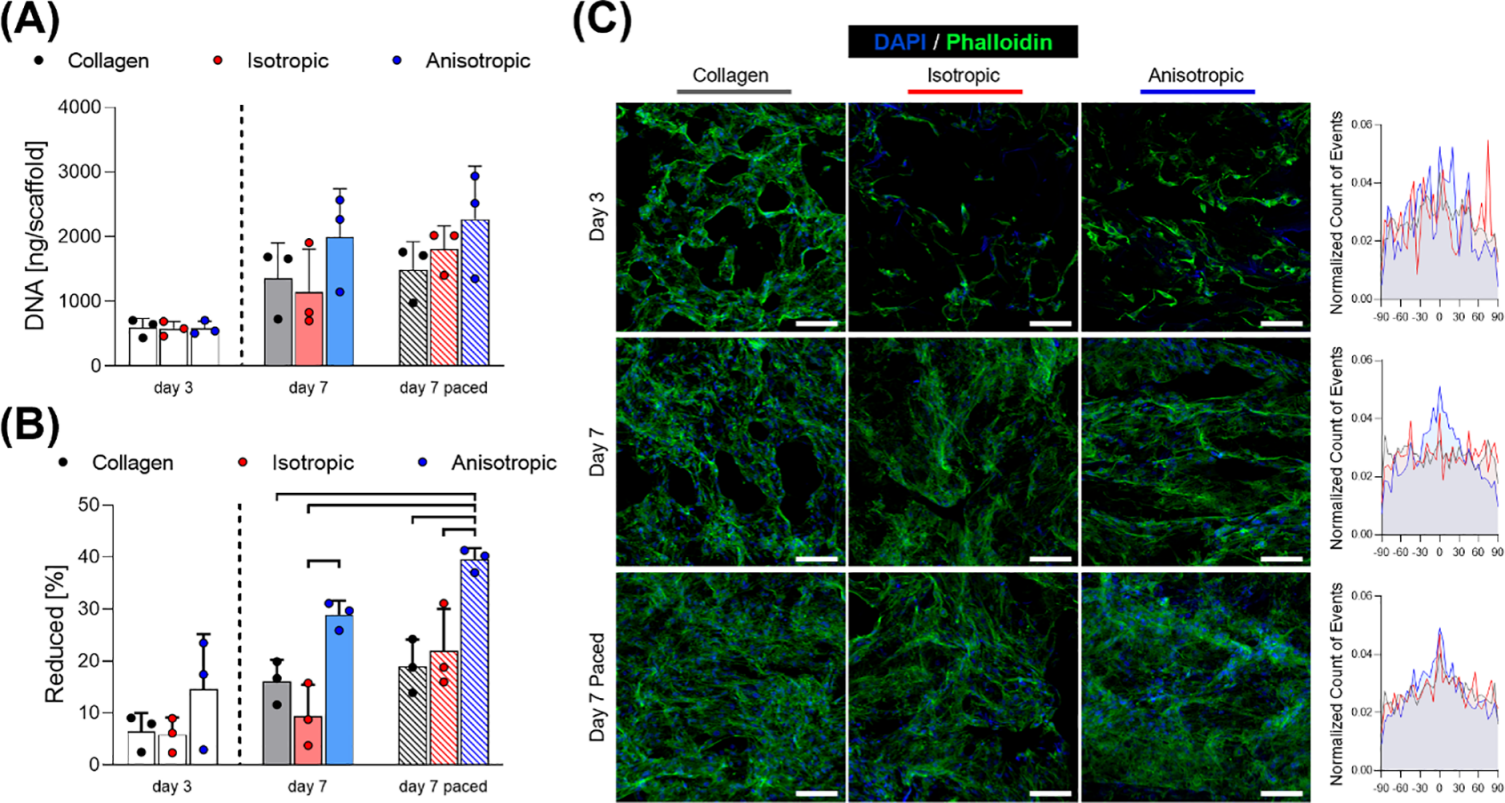
Effects of pacing C3H10 cells in scaffolds on the cell proliferation, metabolism, and orientation. (A) Quantification of DNA via Picogreen assay, expressed as ng per scaffold (*n* = 3). (B) AlamarBlue assay performed on scaffolds (*n* = 3). (C) Micrographs from confocal microscope fluorescent staining for nuclei/f-actin (DAPI and Phalloidin, respectively) and distribution of nuclei orientation. Scale bars: C = 100 μm. Bar graphs demonstrate the mean with error bars representing standard deviation. Data values are presented as associated points. Horizontal lines represent statistical significance (*p* < 0.05) between indicated groups using two-way ANOVA with Tukey’s posthoc test. Data at day 3 have not been compared to the other groups.

In this study, we detail the setup for securing and pacing 3D sponge-like scaffolds, with a custom-made bioreactor to provide electrical pacing; providing detailed drawings and assembly videos to achieve this. As it has been introduced in section 2.7, to promote the adhesion of cultured cells to the scaffolds and implement a level of standardization during assembly of the R3S system, a detailed seeding protocol was optimized. The seeding chambers were fabricated with agarose, and cells were suspended and delivered within a weak Matrigel mix (1:1 ratio mixed with cell suspension in media) which undergoes a rapid sol/gel transition. The rationale for this seeding is that the assembly of R3S involves a compression maneuver by the elastomeric bars on the scaffolds as well as permanent suspension of the scaffolds in a floating and stable position at constant distance from the bottom of the plate. These factors may hinder the cell delivery to the scaffolds and subsequent seeding density. At day 1, DNA content between groups seeded with C3H10 with standard media in a PDMS mold or in R3S was comparable, and no statistical difference was found between the different scaffold types, confirming that the use of the Matrigel mix (1:1) led to a successful seeding yield (Figure S6). Samples seeded with C3H10 in R3S were then studied up to 7 days, applying electrical pacing with this in-house bioreactor and compared to day 3. Electrical cues have the potential to ameliorate cell performance and influence differentiation fate; however, the application of an improper electrical signal can drive uneven electric field distribution, excess of ions with generation of byproducts, all eventually leading to detrimental effect on cell viability.^35^ Pacing patterns, pulse duration, peak intensity, pulse frequency, and duration of the pacing session are all key players in the effective delivery of pacing.^36^ In particular, the adoption of a biphasic pulse instead of monophasic has shown to reduce the risk of byproducts production by limiting the formation of nonreversible-Faradaic reactions.^12^ The choice of a biphasic pulsatile stimulation and 1 h long training sessions have been implemented with a conservative approach to preserve cell wellbeing and prevent excessive production of potentially harmful redox oxygen species. However, in future studies, these parameters can be tested further and adapted according to cell and tissue type. The findings presented here are proof that such choices successfully maintained cell viability and provide sufficient signaling cues to obtain a measurable effect on their activity. As demonstrated by quantification of live/dead imaging, fibroblasts remained viable at both day 3 and 7, with no difference between the collagen alone group, or PEDOT:PSS-based sponges, with or without electrical pacing (Figure 4). From the mosaic micrographs, cells were clearly distributed homogeneously across the scaffolds, with the exception of the terminal regions corresponding to the sites of anchorage with the elastomeric bars. Here, the elastomeric bars held the scaffold ends without breaking them, and by day 7 plastic compression of the scaffolds occurred together with less cell presence. The use of elastomeric bars is meant to not only simplify the assembly sequence but also maintain scaffolds in a free-floating location in the media. Moreover, acting as flexible constraints with known stiffness, these bars are particularly important for application of this rig with mechanically dynamic and contracting cells such as those from the cardiac or skeletal muscles. Moreover, due to the use of PDMS, the stiffness of these bars can also be increased or decreased by several orders of magnitude just by changing the ratio of the two PDMS types (i.e., Sylgard 184 and Sylgard 527),^37^ hence providing the possibility to tailor the system to the specific mechanical requirements of the adopted cell and tissue types. Indeed, monitoring and quantification of the beating of contractile-type cells are crucial toward the understanding of their physiology. Moreover, the platform herein introduced can allow for real-time monitoring during experiments and will enable the indirect quantification of the in vitro models, for example, to determine the frequency of contraction and establish the generated twitch forces^38^ (Figure 1B-iii). DNA quantification verified cell proliferation within all groups, with trends of increased proliferation for paced groups compared with controls (Figure 5.A). Similar results were obtained for the analysis of cell metabolism; in particular, cells on paced anisotropic scaffolds demonstrated significant increases in metabolism compared to all collagen and isotropic groups, suggesting a benefit to cell performance by the synergistic effect of an anisotropic morphology and the external pacing (Figure 5.B).

In terms of cell morphology, no major qualitative difference was observed; when focusing on cell orientation as it related to nuclei orientation, it could be observed that cells aligned along the lamellae of the anisotropic scaffolds already from day 3, and they maintained such directionality up to day 7 (Figure 5.C). As anticipated, cells on collagen or isotropic substrates did not have any preferential alignment at either day 3 or at day 7 without pacing; however, this alignment adopted preferentially in parallel to the electric field of those samples that were subject to electrical pacing. While more studies are necessary to further validate these findings, here it is confirmed that orientation of C3H10 cell line can be influenced by either scaffold geometry or external pacing and indeed this bioreactor system facilitates this, similar to other cell types such as neural stem cells PC-12^39^ and H9C2 rat cardiomyoblast.^40^ Such alignment is hypothesized to derive from mechanisms that include voltage-gated ion channels, G-protein coupling receptors, integrins, cell polarization, and endogenous electric fields.^41−43^

## 4 Conclusions

In this work, we detail the design, fabrication, and validation of an electrical pacing bioreactor and a Rig for Stimulation of Sponge-like Scaffolds (R3S) which can be adopted for real time monitoring of these such systems. These can be manufactured inexpensively and are scalable using rapid prototyping. Electric-field stimulation *in vitro* platforms are becoming increasingly pursued by researchers, and there is a need for systems that expand the repertoire of scaffolds and materials that can be incorporated.

This system is not just limited to use with 3D porous scaffolds, and the electrical pacing component of the system alone can be used to pace monolayer cultures, and the optical accessibility of the system enables assessment of chemotaxis or electrotaxis,^44^ which can be further exploited for live-cell tracking, although this was not the object of the current manuscript. This bioreactor has been designed to deliver equal stimulation to all six wells of a standard cell culture 6-well plate, while specific in silico modeling of the voltage density across the plate is the foundation of a separate future publication. We present an assembly sequence that is suitable for scaffolds generated with multiple material types, with either isotropic or anisotropic morphology and potentially different manufacturing techniques. The impact and validation of this platform is clear. When external pacing was applied to C3H10 cells, no detrimental effect on cell viability was observed, while a synergistic increase in metabolism and cell orientation was found with the combination of anisotropic scaffolds and pacing. Pacing was instrumental in cell alignment whether scaffolds were either electrically insulant (i.e., collagen-based) or conductive (i.e., PEDOT-based), isotropic or anisotropic. This system opens up great opportunity to study the interplay between cells and a wider repertoire of biomaterials that are prefabricated and predesigned, while observing the movement and contraction of these cellularized tissues in vitro.

## Supporting Information Available

Figure S1: Drawings of the components of the mold for the embedding of PDMS on carbon bars for the development of electrodes. Figure S2: Drawing of the lid adapter of the bioreactor. Figure S3: Electrical circuit schematic of the Pacing Bioreactor. Figure S4: Drawing of R3S. Figure S5: Drawings of Mold for Seeding chambers and of a single seeding chamber. Figure S6: Effects of seeding technique of C3H10 cells on scaffolds on cell proliferation (PDF)
Video 1 - Fabrication of electrodes (MP4)
Video 2 - Assembly of bioreactor (MP4)
Video 3 - R3S overview (MP4)
Video 4 - Fabrication of seeding chamber (MP4)
Video 5 - Assembly of scaffolds into chambers and seeding (MP4)
Video 6 - Coupling R3S with seeding chamber (MP4)
Video 7 - Final setup (MP4)
Compressed folder: 01_ElastomericBars_Mold_BOTTOM.STEP 02_ElastomericBars_Mold_MIDDLE.STEP 03_ElastomericBars_Mold_TOP.STEP 04_ElectrodesEmbedding_Mold_BOTTOM.STEP 05_ElectrodesEmbedding_Mold_TOP.STEP 06_LidAdapter.STEP 07_R3S.STEP 08_SeedingChambers_Mold.STEP (ZIP)

## Abbreviations

3D: three-dimensional
DAPI: 4′,6-diamidine-2′-phenylindoledihydrochloride
EDC: *N*-(3-dimethylaminoproypl)-*N*-ethylcarbodiimide hydrochloride
GOPS: glycidoxipropyl-trimethoxysilane
NHS: *N*-hydroxysuccinimide
PDMS: polydimethylsiloxane
PEDOT: PSS, poly(3,4-ethylenedioxythiophene):polystyrene sulfonate
R3S: rig for stimulation of sponge-like scaffolds
ROI: region of interest
